# Supplementary material for: Risk of SARS-CoV-2 infection and hospitalization in individuals with natural, vaccine-induced and hybrid immunity: a retrospective population-based cohort study from Estonia
Source: Sci Rep. 2023 Nov 21;13:20347. doi: 10.1038/s41598-023-47043-6 (PMC10663482; doi:10.1038/s41598-023-47043-6)
Supplement: Supplementary file 1 — Supplementary Information. [file 41598_2023_47043_MOESM1_ESM.pdf]

Supplement

Content

Statistical analysis.

Table S1. Risk of SARS-CoV-2 infection in Delta and Omicron periods, Cohort 1 (natural vs no-immunity)

Table S2. Risk of SARS-CoV-2 infection in Delta end Omicron periods, Cohort 2 (hybrid vs natural immunity)

Table S3. Risk of SARS-CoV-2 infection in Delta end Omicron periods, Cohort 3 (vaccine vs natural immunity)

Table S4. Adjusted HR-sFactors associated with SARS-CoV-2 infection (adjusted HRs together with 95% CI), Estonia 2020–2022.

Table S5. Factors associated with SARS-CoV-2 infection (adjusted HRs together with 95% CI), Estonia 2020–2022.

Statistical analysis

We analysed data from two time periods: from the start of follow-up until 19 December 2021, when the Delta variant was the predominant circulating SARS-CoV-2 strain (proportion of sequenced strains 93%); and 20 December 2021 to 23 February 2022 (end of follow-up), when the Omicron variants (BA1, BA2 and their sublineages) were the predominant strains (proportion of sequenced strains 88%).[21]

We used frequencies and proportions for categorical variables, means and standard deviations (SD) for age, and median and range for baseline date to characterize the study cohorts (Table 1). The follow-up duration is presented in months. The number of confirmed infections and the crude incidence rates (IRs) per 100 person-years were counted for each cohort (Table S1-S3). Cumulative Kaplan–Meier curves are presented to describe SARS-CoV-2 infections in cohorts by different subcohorts (Figure 2).

We performed Cox regression with the SARS-CoV-2 infection or COVID-19 hospitalization as the dependent variable and sex, age group (18-49, 50-64, 65-79, 80+ years), education (higher, < higher), nationality (Estonian, other), CCI score, time (in months) since the last conferring event and number of conferring events, and SARS-CoV-2 testing intensity as independent variables. Multivariable-adjusted hazard ratios (aHRs) and their 95% confidence intervals (CIs) are presented (Tables 2-4).

We developed separate regression models using the same set of independent and dependent variables for the Delta period and the Omicron period. Note that the calendar time affects the hazards of the different cohorts only via the time-dependence of the SARS-CoV-2 exposure risk (background infection rate).

To assess change in the risk of outcomes (and to fulfil the hazards proportionality assumption), we used piecewise hazards models, whereby follow-up time was split into segments (with a 2-month duration), and hazard ratios were calculated separately for each time period by applying a full Cox model (as described above) within each time segment (this was done for both the Delta and Omicron periods).

For Cohorts two and three, based on the matching procedure, weights were assigned to individuals proportional to the number of unexposed individuals matched to each exposed subject. The weights were used in Cox regression models.

A p value of less than 0.05 was considered to indicate statistical significance in all analyses. Data analysis was performed with the statistical software Stata 17.0, and the CCI was calculated using the macro *charlson*.

Table S1. Risk of SARS-CoV-2 (re)infection in Delta end Omicron periods cohort 1 (natural vs no-immunity)

	Delta period				Omicron period			
	**natural**		**no-imm**		**natural**		**no-imm**	
	Number of events	IR per 100 person-year (95% CI)	Number of events	IR per 100 person-year (95% CI)	Number of events	IR per 100 person-year (95% CI)	Number of events	IR per 100 person-year (95% CI)
Full follow-up period	520	3.8 (3.5,4.1)	3507	20.1 (19.5,20.8)	2778	136.4 (131.4,141.6)	2716	85.3 (82.2,88.6)
Hospitalized	14	0.09 (0.05,0.15)	255	1.23 (1.09,1.39)	5	0.25 (0.1,0.59)	57	1.79 (1.38,2.32)
Age groups								
18–49 years	388	4.6 (4.2,5.1)	2404	24.2 (23.2,25.2)	2075	168.2 (161.1,175.6)	1926	109.8 (105,114.8)
50–64 years	70	2.1 (1.7,2.7)	747	17.5 (16.3,18.8)	536	110 (101.1,119.7)	592	74 (68.3,80.2)
65–79 years	38	2.4 (1.8,3.4)	247	11.3 (9.9,12.8)	143	64.3 (54.5,75.7)	153	35.7 (30.5,41.8)
80+ years	24	4.1 (2.7,6.1)	109	10.7 (8.9,13.)	24	25.7 (17.3,38.4)	45	22.5 (16.8,30.2)
Sex								
Male	214	3.5 (3.0,4.0)	1384	17.8 (16.8,18.7)	1183	131.9 (124.6,139.7)	1062	74.6 (70.3,79.3)
Female	306	4.0 (3.6,4.5)	2123	22 (21.1,23.0)	1595	139.9 (133.2,146.9)	1654	94 (89.6,98.6)
Education								
below higher	393	3.9 (3.6,4.3)	2660	21.3 (20.5,22.1)	2016	133.9 (128.2,139.9)	2050	91.8 (87.9,95.8)
higher	115	3.4 (2.9,4.1)	772	21.2 (19.8,22.8)	723	158.1 (147,170.1)	613	92.8 (85.7,100.4)
unknown	12	2.8 (1.6,4.9)	75	5.8 (4.6,7.2)	39	53.1 (38.8,72.6)	53	18.4 (14,24.1)
Nationality								
estonian	249	4.4 (3.0.9,5)	2227	25.2 (24.2,26.2)	1196	147 (138.9,155.5)	1570	99.5 (94.7,104.6)
else	260	3.3 (2.0.9,3.7)	1230	16.1 (15.2,17)	1547	132.1 (125.6,138.8)	1100	78.6 (74.1,83.4)
unknown	11	3.7 (2,6.6.0)	50	5.4 (4.1,7.1)	35	68 (48.8,94.7)	46	22.3 (16.7,29.8)
Charlson score								
0	447	3.8 (3.4,4.1)	3174	20.3 (19.6,21)	2506	143.8 (138.3,149.5)	2481	86.4 (83.1,89.9)
1–2	57	3.7 (2.8,4.7)	257	17.7 (15.7,20.0)	234	100.9 (88.7,114.7)	214	82.9 (72.5,94.8)
3+	16	3.8 (2.3,6.2)	76	24.1 (19.3,30.2)	38	61.5 (44.7,84.5)	21	39.2 (25.5,60.1)

Intensity of testing per 100 days								
<1	225	2.1 (1.8,2.4)	2469	16 (15.4,16.6)	1679	105.3 (100.3,110.4)	1985	68.1 (65.2,71.2)
1..<2	233	10.1 (8.9,11.5)	779	52.7 (49.1,56.5)	853	262.9 (245.9,281.2)	537	263.7 (242.3,287.0)
2+	62	8.6 (6.7,11.1)	259	50.1 (44.4,56.6)	246	210.3 (185.6,238.3)	194	296.6 (257.7,341.5)
Time since the last immunity conferring event								
<100 days	68	2.9 (2.3,3.6)	530	20.3 (18.6,22.1)	378	119.2 (107.7,131.8)	401	84.2 (76.3,92.8)
100-199 days	339	3.6 (3.0.3,4)	2470	20 (19.2,20.8)	1909	138.5 (132.5,144.9)	1908	84.5 (80.8,88.4)
≥200 days	113	5.5 (4.0.6,6.6)	507	20.7 (18.9,22.5)	491	143.8 (131.6,157.1)	407	90.8 (82.4,100)
No of immunity conferring events								
1	518	3.8 (3.5,4.1)	3479	20.1 (19.5,20.8)	2728	137.2 (132.1,142.4)	2692	85.4 (82.2,88.6)
≥2	2	1 (0.2,3.9)	28	17.9 (12.3,25.9)	50	104.3 (79.1,137.6)	24	83 (55.7,123.9)

Table S2. Risk of SARS-CoV-2 (re)infection in Delta end Omicron periods cohort 2 (hybrid vs natural)

	Delta period				Omicron period			
	**hybrid**		**natural**		**hybrid**		**natural**	
	Number of events	IR per 100 person-year (95% CI)	Number of events	IR per 100 person-year (95% CI)	Number of events	IR per 100 person-year (95% CI)	Number of events	IR per 100 person-year (95% CI)
Full follow-up period	149	1.5 (1.3,1.8)	271	3.9 (3.4,4.4)	1250	115.8 (109.5,122.3)	1340	130.5 (123.7,137.7)
Hospitalized					1	0.09 (0.01,0.66)	4	0.39 (0.15,1.04)
Age groups								
18–49 years	86	1.7 (1.4,2.1)	173	4.8 (4.1,5.6)	920	144.2 (135.2,153.8)	892	168.6 (157.9,180)
50–64 years	29	1 (0.7,1.5)	53	2.7 (2.1,3.5)	265	87.9 (78,99.2)	339	116.3 (104.6,129.4)
65–79 years	24	1.8 (1.2,2.6)	24	2.4 (1.6,3.6)	45	41.7 (31.1,55.8)	90	63.3 (51.5,77.8)
80+ years	10	2.1 (1.1,3.9)	21	5.1 (3.3,7.8)	20	61.6 (39.7,95.4)	19	29.7 (18.9,46.5)
Sex								
Male	62	1.4 (1.1,1.8)	100	3.2 (2.6,3.9)	567	113.8 (104.8,123.6)	575	128.3 (118.3,139.3)
Female	87	1.6 (1.3,2.0)	171	4.4 (3.8,5.1)	683	117.4 (108.9,126.5)	765	132.2 (123.2,141.9)
Education								
below higher	88	1.5 (1.2,1.9)	206	4.1 (3.5,4.6)	736	110.5 (102.8,118.8)	981	129.3 (121.4,137.6)
higher	52	1.4 (1.1,1.8)	57	3.4 (2.6,4.5)	492	126.8 (116.1,138.5)	340	150.4 (135.2,167.2)
unknown	9	2.7 (1.4,5.1)	8	3.2 (1.6,6.3)	22	85.8 (56.5,130.3)	19	45.7 (29.1,71.6)
Nationality								
estonian	96	1.5 (1.2,1.8)	127	4.6 (3.9,5.5)	790	118.9 (110.9,127.4)	551	139.3 (128.1,151.4)
else	52	1.6 (1.2,2.1)	137	3.3 (2.8,3.9)	441	110.5 (100.6,121.3)	775	127.3 (118.7,136.6)
unknown	1	0.8 (0.1,5.7)	7	5.3 (2.5,11.1)	19	118.1 (75.3,185.1)	14	62.3 (36.9,105.1)
Charlson score								
0	113	1.4 (1.2,1.7)	213	3.7 (3.3,4.3)	1127	120.5 (113.6,127.7)	1178	140.2 (132.4,148.4)
1–2	25	1.9 (1.3,2.8)	47	5 (3.7,6.6)	104	86.9 (71.7,105.4)	135	97.2 (82.1,115.0)
3+	11	3.1 (1.7,5.7)	11	3.3 (1.0.8,6)	19	76.9 (49.1,120.6)	27	56.9 (39.0,83.0)
Intensity of testing per 100 days								

<1	90	1 (0.8,1.3)	128	2.2 (1.9,2.6)	931	97.2 (91.2,103.7)	876	103.3 (96.7,110.4)
1– <2	41	5.4 (4,7.4.0)	86	10.9 (8.9,13.5)	245	265.4 (234.1,300.8)	308	255.1 (228.2,285.3)
2+	18	7.7 (4.8,12.2)	57	14.4 (11.1,18.7)	74	245.4 (195.4,308.1)	156	268.4 (229.4,313.9)
Time since the last immunity conferring event								
<100 days	120	1.3 (1.1,1.6)	27	3 (2.1,4.4)	1183	115.5 (109.1,122.3)	127	113.6 (95.4,135.1)
100-199 days	29	3.4 (2.3,4.9)	180	3.6 (3.1,4.2)	67	119.4 (94.0,151.7)	945	129.3 (121.3,137.8)
≥200 days			64	5.8 (4.5,7.4)			268	145.7 (129.3,164.2)
No of immunity conferring events								
1	149	1.5 (1.3,1.8)	270	3.9 (3.4,4.4)	1250	115.8 (109.6,122.4)	1333	130.2 (123.4,137.4)
≥2			1	4.9 (0.7,34.7)			7	260.9 (124.4,547.3)

Table S3. Risk of SARS-CoV-2 (re)infection in Delta end Omicron periods cohort 3 (vaccine vs natural)

	Delta period				Omicron period			
	**vaccine**		**natural**		**vaccine**		**natural**	
	Number of events	IR per 100 person-year (95% CI)	Number of events	IR per 100 person-year (95% CI)	Number of events	IR per 100 person-year (95% CI)	Number of events	IR per 100 person-year (95% CI)
Full follow-up period	5519	13.1 (12.7,13.4)	1265	3.3 (3.2,3.5)	3191	116.6 (112.6,120.7)	1265	3.3 (3.2,3.5)
Hospitalized	140	0.33 (0.28,0.39)	31	0.08 (0.06,0.12)	14	0.51 (0.3,0.8)	18	0.32 (0.2,0.51)
Age groups								
18–49 years	2493	18 (17.4,18.8)	692	4.5 (4.2,4.8)	1903	175.9 (168.2,184)	692	4.5 (4.2,4.8)
50–64 years	1700	13.3 (12.7,14)	221	2.2 (2.0,2.6)	956	106.3 (99.8,113.3)	221	2.2 (2.0,2.6)
65–79 years	948	8.2 (7.7,8.8)	216	2.4 (2.1,2.7)	270	48.7 (43.2,54.8)	216	2.4 (2.1,2.7)
80+ years	378	9 (8.2,10.0)	136	3.9 (3.3,4.6)	62	30.8 (24.0,39.5)	136	3.9 (3.3,4.6)
Sex								
Male	2271	12.7 (12.2,13.3)	499	3.1 (2.9,3.4)	1333	111.9 (106,118)	499	3.1 (2.9,3.4)
Female	3248	13.3 (12.8,13.8)	766	3.5 (3.2,3.7)	1858	120.2 (114.8,125.8)	766	3.5 (3.2,3.7)
Education								
below higher	3068	11.9 (11.5,12.3)	963	3.5 (3.2,3.7)	1981	106.3 (101.7,111.1)	963	3.5 (3.2,3.7)
higher	2195	15.5 (14.9,16.2)	252	3 (2.6,3.4)	1144	151.9 (143.4,161)	252	3 (2.6,3.4)
unknown	256	10.9 (9.7,12.3)	50	3.2 (2.4,4.2)	66	54.4 (42.7,69.2)	50	3.2 (2.4,4.2)
Nationality								
estonian	4492	13.6 (13.2,14.0)	569	4.1 (3.8,4.4)	2333	117.9 (113.2,122.7)	569	4.1 (3.8,4.4)
else	978	10.9 (10.3,11.6)	677	2.9 (2.7,3.1)	825	113.6 (106.1,121.7)	677	2.9 (2.7,3.1)
unknown	49	13.2 (10.0,17.5)	19	3.5 (2.2,5.4)	33	102.7 (73,144.4)	19	3.5 (2.2,5.4)
Charlson score								
0	4428	13.4 (13.0,13.8)	928	3.2 (3,3.5)	2810	125.3 (120.7,130)	928	3.2 (3.0,3.5)
1–2	868	11.6 (10.9,12.4)	244	3.6 (3.2,4.1)	332	80.8 (72.5,89.9)	244	3.6 (3.2,4.1)
3+	223	13.1 (11.5,15.0)	93	3.5 (2.9,4.3)	49	59.1 (44.6,78.2)	93	3.5 (2.9,4.3)
Intensity of testing per 100 days								
<1	3550	9.2 (8.9,9.5)	1010	2.8 (2.6,3)	2429	97 (93.2,100.9)	1010	2.8 (2.6,3.0)

1– <2	1472	53.2 (50.6,56.0)	179	14.7 (12.7,17.1)	619	336.5 (311.0,364.0)	179	14.7 (12.7,17.1)
2+	497	60.5 (55.5,66.1)	76	12.7 (10.2,16)	143	290.4 (246.5,342.1)	76	12.7 (10.2,16.0)
Time since the last immunity conferring event								
<100 days	4570	12.2 (11.9,12.6)	179	3.1 (2.7,3.6)	3009	115.9 (111.9,120.1)	179	3.1 (2.7,3.6)
100-199 days	949	19.7 (18.4,20.9)	815	3.1 (2.9,3.3)	182	128.2 (110.9,148.3)	815	3.1 (2.9,3.3)
≥200 days			271	4.7 (4.2,5.3)		116.7 (112.7,120.8)	271	4.7 (4.2,5.3)
No of immunity conferring events								
1	5491	13 (12.7,13.4)	1254	3.3 (3.1,3.5)	3189	36.6 (9.1,146.2)	1254	3.3 (3.1,3.5)
≥2	28	16.1 (11.1,23.3)	11	7.1 (3.9,12.7)	2		11	7.1 (3.9,12.7)

Table S4. Factors associated with SARS-CoV-2 infection (adjusted HRs together with 95% CI), Estonia 2020–2022.

	**COHORT 1: NATURAL VS NO-IMM**		**COHORT 2: HYBRID VS NATURAL**		**COHORT 3: VACCINE VS NATURAL**	
	**Delta**	**Omicron**	**Delta**	**Omicron**	**Delta**	**Omicron**
	adjHR (95% CI)	adjHR (95% CI)	adjHR (95% CI)	adjHR (95% CI)	adjHR (95% CI)	adjHR (95% CI)
Exposed vs unexposed	0.17*** (0.15,0.19)	1.26*** (1.19,1.33)	0.61*** (0.46,0.80)	1.05 (0.93,1.19)	4.90*** (4.48,5.36)	1.13*** (1.06,1.21)
Sex						
Female vs male	1.21*** (1.14,1.29)	1.18*** (1.12,1.25)	1.25* (1.03,1.53)	1.05 (0.97,1.14)	1.03 (0.98,1.08)	1.12*** (1.08,1.17)
Age groups						
50–64 vs <50 years	0.72*** (0.67,0.78)	0.67*** (0.63,0.72)	0.62*** (0.48,0.80)	0.68*** (0.62,0.75)	0.82*** (0.78,0.87)	0.68*** (0.64,0.71)
65–79 vs <50 years	0.50*** (0.44,0.57)	0.37*** (0.33,0.42)	0.72 (0.51,1.02)	0.37*** (0.31,0.45)	0.58*** (0.54,0.63)	0.38*** (0.35,0.41)
80+ vs <50 years	0.48*** (0.40,0.57)	0.19*** (0.15,0.25)	0.83 (0.53,1.30)	0.26*** (0.19,0.37)	0.60*** (0.54,0.67)	0.17*** (0.14,0.19)
Nationality						
estonian vs else	0.68*** (0.63,0.72)	0.91*** (0.86,0.96)	0.80* (0.65,0.98)	0.97 (0.90,1.05)	0.78*** (0.73,0.82)	1.04 (0.99,1.08)
Education						
higher vs below higher	0.85*** (0.79,0.92)	0.92* (0.87,0.98)	0.73** (0.58,0.91)	0.99 (0.91,1.07)	1.03 (0.98,1.09)	1.11*** (1.06,1.16)
Charlson score						
1–2 vs 0	1.12 (1.00,1.27)	1.16** (1.05,1.29)	1.54** (1.16,2.06)	1.03 (0.89,1.18)	1.12*** (1.05,1.20)	0.98 (0.92,1.05)
3+ vs 0	1.62*** (1.31,2.01)	0.8 (0.62,1.04)	1.38 (0.85,2.26)	0.89 (0.66,1.21)	1.26*** (1.12,1.42)	0.80** (0.70,0.92)
Intensity of testing per 100 days						
1–<2 vs <1	3.21*** (2.98,3.46)	2.80*** (2.62,2.98)	4.57*** (3.65,5.71)	2.39*** (2.16,2.63)	5.04*** (4.75,5.34)	2.37*** (2.22,2.53)
2+ vs <1	3.21*** (2.86,3.61)	2.98*** (2.69,3.31)	6.11*** (4.67,7.99)	2.55*** (2.22,2.94)	6.15*** (5.63,6.72)	2.29*** (2.06,2.54)
Time from last immunization (natural or vaccine) per month	1.03*** (1.01,1.04)	1.02** (1.00,1.03)	1.09*** (1.05,1.14)	1.01 (0.99,1.03)	1.13*** (1.12,1.15)	1.01** (1.00,1.02)

adjHR – adjusted to all variables in table

* p<0.05, ** p<0.01, *** p<0.001

Table S5. Factors associated with COVID-19 hospitalization (adjusted HRs together with 95% CI), Estonia 2020–2022

	Cohort 1: Natural vs no-imm		Cohort 2: Hybrid vs natural		Cohort 3: Vaccine vs natural	
	**Delta** adjHR (95% CI)	**Omicron** adjHR (95% CI)	**Delta** adjHR (95% CI)	**Omicron** adjHR (95% CI)	**Delta** adjHR (95% CI)	**Omicron** adjHR (95% CI)
Exposed vs unexposed	0.05*** (0.03,0.11)	0.10*** (0.04,0.26)		0.7 (0.04,12.70)	7.19*** (4.02,12.84)	2 (0.64,6.25)
Sex						
Female vs male	0.59*** (0.44,0.77)	0.43** (0.26,0.72)	2.42 (0.26,22.65)	0.29 (0.05,1.88)	0.58*** (0.43,0.79)	0.59 (0.29,1.21)
Age groups						
50–64 vs <50 years	3.13*** (2.08,4.70)	4.84*** (2.08,11.26)		0.71 (0.06,8.97)	2.20* (1.20,4.05)	1.13 (0.33,3.84)
65–79 vs <50 years	5.82*** (3.81,8.90)	8.82*** (3.71,20.96)	0.7 (0.03,17.27)	0 (0.00,.)	3.50*** (1.93,6.34)	1.61 (0.48,5.44)
80+ vs <50 years	9.29*** (5.83,14.80)	15.71*** (6.22,39.65)	1.41 (0.05,37.47)	6.78 (0.52,88.77)	5.95*** (3.19,11.08)	7.13** (2.19,23.21)
Nationality						
estonian vs else	0.77 (0.58,1.01)	1.15 (0.69,1.91)	1.25 (0.19,8.40)	3.05 (0.31,29.89)	0.98 (0.68,1.40)	1.77 (0.80,3.90)
Education						
higher vs below higher	1.15 (0.81,1.64)	0.75 (0.37,1.53)	0.91 (0.09,9.39)	0.67 (0.07,6.32)	0.58* (0.38,0.90)	0.6 (0.21,1.75)
Charlson score						
1–2 vs 0	1.45 (1.00,2.12)	2.20* (1.20,4.01)	2.47 (0.12,50.20)	6.74 (0.71,64.08)	3.21*** (2.22,4.66)	1.19 (0.42,3.33)
3+ vs 0	3.43*** (2.19,5.36)	2.57* (1.07,6.17)	9.89 (0.44,224.57)	0 (0.00,.)	4.80*** (3.07,7.50)	5.29*** (2.07,13.53)
Intensity of testing per 100 days						
1–<2 vs <1	2.73*** (1.86,4.00)	3.34** (1.61,6.91)	3.69 (0.32,42.47)	5.09 (0.44,59.28)	6.02*** (4.03,9.00)	5.05** (1.64,15.52)
2+ vs <1	3.34*** (2.00,5.57)	6.47*** (2.83,14.80)	11.00* (1.48,81.54)	16.93** (2.26,126.86)	12.22*** (7.88,18.96)	10.52*** (3.54,31.24)
Time from last immunization (natural or vaccine) per month	1.03 (0.97,1.10)	1.03 (0.91,1.16)	1.15 (0.85,1.55)	1.09 (0.73,1.63)	1.24*** (1.14,1.34)	0.97 (0.78,1.21)

adjHR – adjusted to all variables in table
* p<0.05, ** p<0.01, *** p<0.001
